# Transient hyponatremia of prematurity caused by mild Bartter syndrome type II: a case report

**DOI:** 10.1186/s12887-020-02214-6

**Published:** 2020-06-26

**Authors:** Subhrata Verma, Rahul Chanchlani, Victoria Mok Siu, Guido Filler

**Affiliations:** 1 Department of Pediatrics, Schulich School of Medicine and Dentistry, University of Western Ontario, 1151 Richmond Street, London, ON N6A5C1 Canada; 2 Division of Pediatric Nephrology, Department of Pediatrics, McMaster Children’s Hospital, McMaster University, 1200 Main Street West, Hamilton, ON L8N 3Z5 Canada; 3 Division of Medical Genetics, and Department of Biochemistry, London Health Sciences Centre, 800 Commissioners Road East, London, ON N6A 5W9 Canada; 4 Children’s Health Research Institute, 750 Baseline Road East, London, ON N6C 2R5 Canada; 5 Departments of Pathology and Laboratory Medicine, Division of Nephrology, Lilibeth Caberto Kidney Clinical Research Unit, London Health Sciences Centre, University of Western Ontario, 800 Commissioners Road East, London, ON N6A 5W9 Canada

**Keywords:** Pediatric nephrology, Neonatology, Genetics, Case report

## Abstract

**Background:**

Bartter syndrome subtypes are a group of rare renal tubular diseases characterized by impaired salt reabsorption in the tubule, specifically the thick ascending limb of Henle’s loop. Clinically, they are characterized by the association of hypokalemic metabolic alkalosis, hypercalciuria, nephrocalcinosis, increased levels of plasma renin and aldosterone, low blood pressure and vascular resistance to angiotensin II. Bartter syndrome type II is caused by mutations in the renal outer medullary potassium channel (ROMK) gene (*KCNJ1*), can present in the newborn period and typically requires lifelong therapy.

**Case presentation:**

We describe a case of a prematurely born female infant presenting with antenatal polyhydramnios, and postnatal dehydration and hyponatremia. After 7 weeks of sodium supplementation, the patient demonstrated complete resolution of her hyponatremia and developed only transient metabolic alkalosis at 2 months of age but continues to be polyuric and exhibits hypercalciuria, without development of nephrocalcinosis. She was found to have two pathogenic variants in the *KCNJ1* gene: a frameshift deletion, p.Glu334Glyfs*35 and a missense variant, p. Pro110Leu. While many features of classic ROMK mutations have resolved, the child does have Bartter syndrome type II and needs prolonged pediatric nephrology follow-up.

**Conclusion:**

Transient neonatal hyponatremia warrants a multi-system workup and genetic variants of *KCNJ1* should be considered.

## Background

Bartter syndrome was initially described in 1962 by Bartter et al. as a renal tubular disorder characterized by hypokalemia, metabolic alkalosis, a low or normal blood pressure and elevated renin [1]. The hyperreninemia and hyperaldosteronism occur due to volume depletion activating the renin-angiotensin-aldosterone system. Bartter syndrome can also involve polyuria, polydipsia, normal to increased urinary calcium excretion, normal or mildly decreased serum magnesium, and occasionally hypophosphatemia. It is categorized into five types each with specific associated mutations and clinical presentations [2, 3].

Variability in Bartter syndrome presentations are seen even within subtypes. A few cases of transient Bartter syndrome have been reported with mutations located in the melanoma-associated antigen D2 (*MAGE-D2*) gene located on the X-chromosome [2, 4, 5]. Bartter syndrome type II (MIM #241200) is caused by mutations in the renal outer medullary potassium channel (ROMK) potassium channel gene (*KCNJ1*) (MIM *600359), can manifest in the neonatal period with hypokalemic metabolic alkalosis, and typically requires lifelong electrolyte supplementation [6]. We are aware of a single case of transient Bartter syndrome in a preterm patient who was compound heterozygous for pathogenic variants in the *KCNJ1* gene: a paternally inherited Arg338Stop variant and a maternally inherited Met357Thr variant [7]. The patient’s symptoms resolved by age three after 1 year of indomethacin treatment, possibly fitting the diagnosis of transient Bartter syndrome.

Here we describe another patient with compound heterozygous variants in the *KCNJ1* gene, who exhibited the features of neonatal Bartter syndrome but showed spontaneous resolution of electrolyte abnormalities by 3 months of age while continuing to have polyuria and hypercalciuria.

## Case presentation

This female patient was born at 33 weeks and 6 days to non-consanguineous, Caucasian parents by spontaneous vaginal delivery. The Apgar scores were 6 and 7 at one and 5 min of life, respectively, and the birth weight was 1450 g (5th percentile) which would be classified as very low birth weight (< 1500 g). The antenatal ultrasound demonstrated polyhydramnios and a possible duplex collecting system without hydronephrosis. The remainder of the pregnancy was uneventful with no gestational diabetes. She required positive pressure ventilation at birth and was admitted to the neonatal intensive care unit with poor responsiveness and requirement of non-invasive ventilation. On day of life 7 she was thought to be clinically dehydrated and had a 22% weight loss since birth, however her blood pressures remained within normal limits at 74/41 mmHg.

### Investigations

**Fig. 1:**
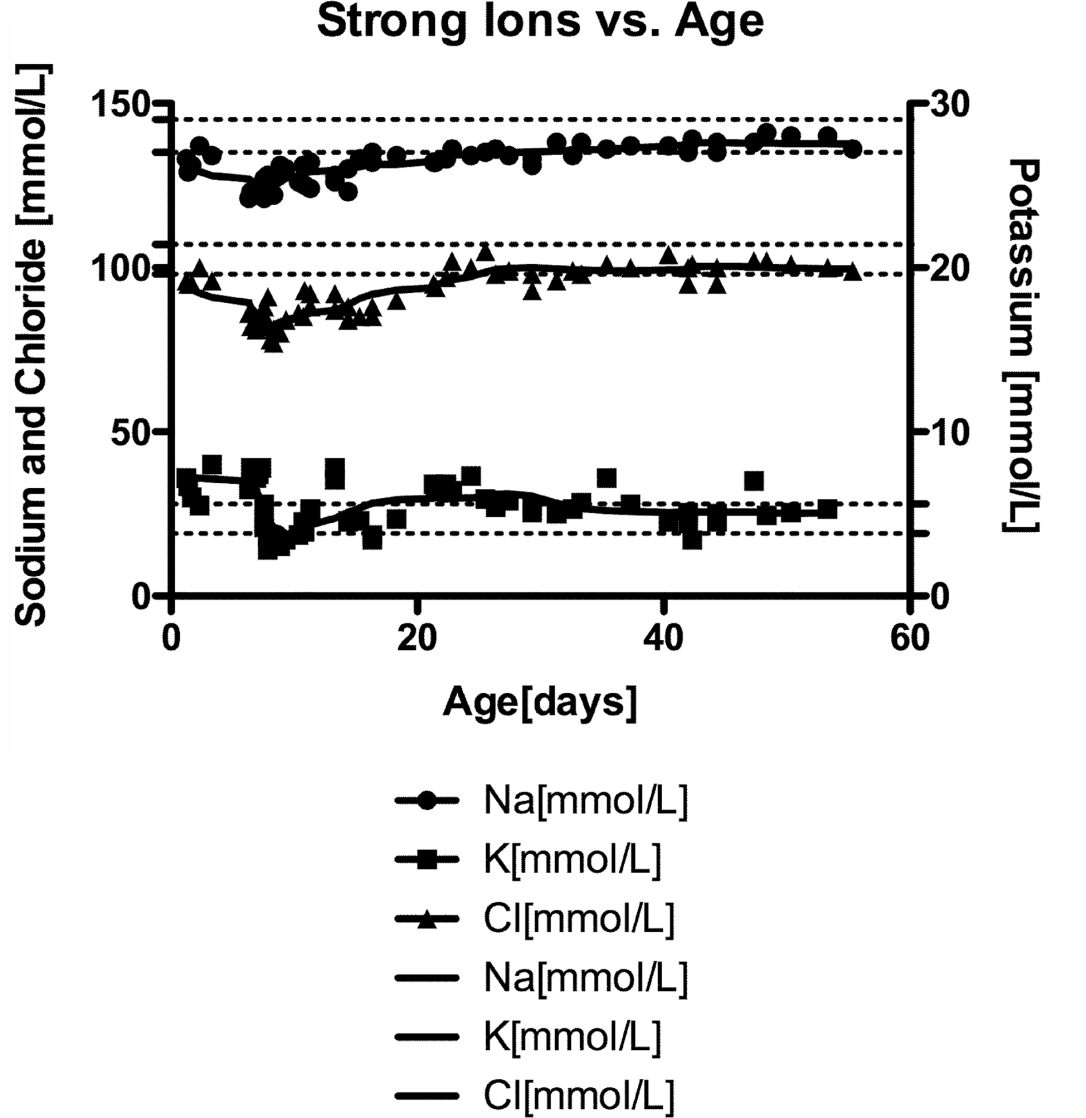
Strong Ions vs Age. Trend of strong ions (sodium and potassium) from point of care blood gases and serum electrolytes overtime from day of life 0 until 7 weeks of age. The straight lines represent the serum electrolyte values, and the shapes represent point of care test values

The initial gas done at 15 h of life showed a pH of 7.26 [normal 7.35–7.45], pCO2 of 59 mmHg [normal 35–45 mmHg], bicarbonate of 28.3 mmol/L [normal 22–26 mmol/L] and base excess of − 2.7 mmol/L [normal − 2 – + 3 mmol/L] (Fig. 1). This represents a respiratory acidosis. Repeated capillary blood gases over the next 7 days showed hyponatremia ranging between 121 and 137 mmol/L [normal 135–145 mmol/L], hyperkalemia ranging between 5.5–8.0 mmol/L [normal for age 4.0–6.5 mmol/L] in both mildly hemolyzed and non-hemolyzed samples, and chloride values ranging between 86 and 100 mmol/L [normal 98–107 mmol/L]. These electrolyte abnormalities are in keeping with early Bartter syndrome type II. By day of life 7 the sodium, potassium and chloride levels decreased to 121 mmol/L, 2.8 mmol/L, and 82 mmol/L, respectively, on an arterial blood gas. At this time, the urea was 19 mmol/L (normal for age < 7 mmol/L) and creatinine was 52 μmol/L (normal for age < 53 μmol/L). A cystatin C eGFR done at 7 weeks of age was 58 mL/min [normal for age, reference intervals not well established]. A post-natal renal ultrasound did not reveal medullary nephrocalcinosis.

Further investigations revealed a normal magnesium level of 0.95 mmol/L [0.65–1.05 mmol/L], an elevated aldosterone of > 27,000 pmol/L [normal 320–4621 pmol/L], and elevated renin at > 3000 ng/L [upper limit of normal 175 ng/L] [8]. The cortisol was 629 nmol/L [133–537 nmol/L], ACTH was 6.6 pmol/L [normal 1.98–12.47 pmol/L], and 17-OHP was mildly elevated at 14.8 nmol/L [normal 0.2–4.7 nmol/L]. These findings were sufficient to rule out congenital adrenal hyperplasia (CAH) due to 21 hydroxylase deficiency.

Testing of the urine on day of life 7 revealed an osmolality of 369 mOsm/kg [reference interval in neonates not well established], high sodium of 118 mmol/L [renal salt wasting defined as urinary sodium > 40 mmol/L], low potassium of 7 mmol/L, chloride of 94 mmol/L, and low urine creatinine of 0.3 mmol/L. As there are no established normal urine electrolyte values, these should be interpreted in context with serum values. This represents a fractional sodium excretion of 16.9% in a clinically hypovolemic patient which would suggest tubular dysfunction, however, there are no reference intervals for premature infants.

### Differential diagnosis

Initially consideration was given to renal etiologies of primary sodium depletion including Bartter syndrome and pseudohypoaldosteronism. Adrenogenital syndromes were unlikely given normal female genitalia and normal newborn screening results. Of the Bartter syndromes, type II was more likely given that she initially did not present with hypokalemia or hypochloremia. These electrolyte abnormalities can develop later in the presentation of type II Bartter syndrome. Additionally, given the normal blood pressure measurements and lack of metabolic acidosis, pseudohypoaldosteronism type I and II were unlikely.

### Treatment

The patient required sodium chloride supplementation up to a maximum dose of 7.9 mmol/kg/day included in the total parenteral nutrition, started on day of life 7 to gradually achieve normal sodium levels. A trial of fludrocortisone therapy was recommended by endocrinology to definitively rule out CAH. The patient received two doses given on day 9 (0.03 mg/kg/dose) of life and was without clinical response. The patient’s electrolytes normalized by 4 weeks of life and supplementation was gradually weaned off by 7 weeks of life. Her electrolytes have been normal since then, apart from a mildly elevated bicarbonate of 29 mmol/L, which peaked once at 40 mmol/L at 2 months of age but subsequently normalized.

### Molecular investigations

**Fig. 2:**
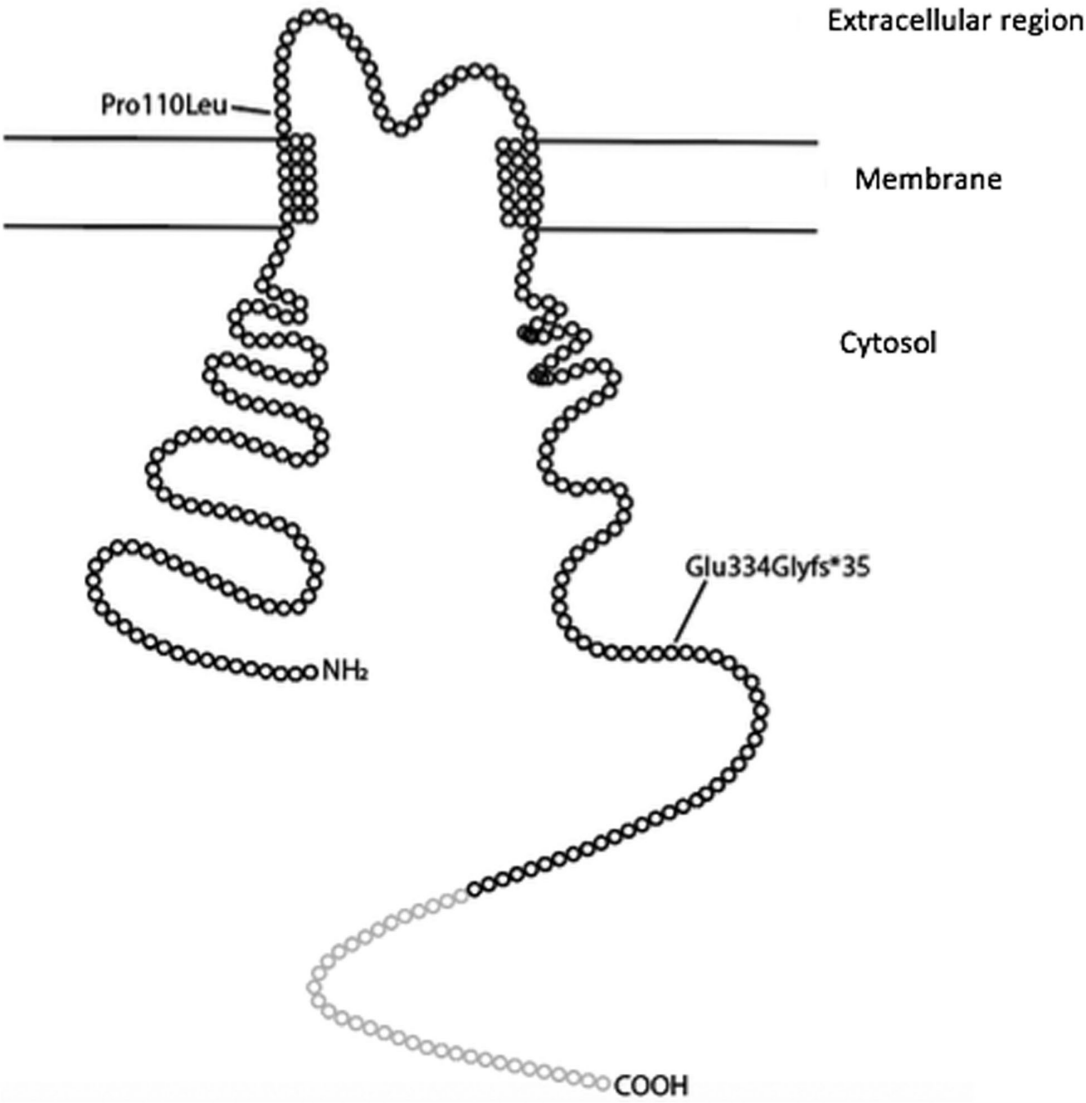
ROMK channel protein 2D structural model. Two mutations are indicated in their approximate location, a missense mutation at Pro110Leu and a frameshift mutation at Glu334Gly with premature protein termination 35 amino acids downstream near the C-terminus

A gene panel associated with Bartter syndrome (including *KCNJ1*, *AP2S1*, *CASR*, *GNA11*, *SLC12A1*, and *SLC12A3*) was performed at a commercial lab (Prevention Genetics, Marshfield, Wisconsin, USA). Results showed two pathogenic variants in the *KCNJ1* gene [1]: a paternally inherited frameshift deletion, p.Glu334Glyfs*35 (c.996_999del, exon 5), predicted to result in premature protein termination and previously reported as causative for Bartter syndrome [6], and [2] a maternally inherited missense variant, p. Pro110Leu (c.329C > T, exon 5) (Fig. 2), previously reported to be pathogenic in compound heterozygosity with other pathogenic variants in *KCNJ1* [8–10].

### Outcome and follow-up

On follow up assessment at 6 months of age, electrolytes remained within normal limits with a sodium of 138 mmol/L, potassium of 4.5 mmol/L, and chloride of 101 mmol/L. Her calcium was slightly elevated at 2.92 mmol/L [normal for age 2.3–2.62 mmol/L], phosphate was 2.31 mmol/L [normal for age 1.55–2.71 mmol/L], and magnesium was 1.00 mmol/L [normal for age [0.65–1.05 mmol/L]. Her Schwartz eGFR at this time was 56 mL/min/1.73m^2^. She had persistent hypercalciuria with a urine calcium of 0.8 mmol/L and a urine creatinine of less than 0.1 mmol/L (ratio > 8), which may resemble residual disease activity. However, the ultrasound at 6 months of age did not show nephrocalcinosis. Repeat venous blood gas at 6 months of age was normal with a pH 7.35, pCO2 52 mmHg and bicarbonate 29 mmol/L. At this time her weight was 6.08 kg (11th percentile) and length was 60.8 cm (2nd percentile). Her Schwartz eGFR was 61 mL/min/1.73 m^2^ which is likely an underestimation as this was a Jaffe-based creatinine in the community. She also had ongoing polyuria and polydipsia. Follow up at 15 months of age showed a normalized renin and aldosterone level at 24.5 ng/L [upper limit of normal 175 ng/L] and 1153 pmol/L [normal for age 112–1575 pmol/L], respectively.

## Discussion and conclusion

This female infant demonstrates a case of Bartter syndrome type II with compound heterozygosity for two different pathogenic *KCNJ1* gene variants who developed resolution of hyponatremia by 7 weeks of age while the classic hypokalemic metabolic alkalosis was not seen on follow up. She had a history of antenatal polyhydramnios and was born prematurely with hyponatremia, hyperkalemia and hyperreninemia, features in keeping with type II Bartter syndrome. By 6 months of age, the electrolyte abnormalities and hyperreninemia had resolved and there was no hypokalemic alkalosis, however, she had ongoing polyuria, polydipsia and hypercalciuria that require ongoing evaluation and management. Although use of a COX-2 inhibitor such as indomethacin has not yet been initiated, this may be considered in her future management.

Hyponatremia in Bartter syndrome occurs due to impaired sodium reabsorption in the thick ascending loop of Henle. This is a consequence of impaired potassium reabsorption in Bartter syndrome type II. The gradient created by the ROMK channel protein normally allows for proper functioning of the Na-K-2Cl, a co-transporter is responsible for sodium reabsorption [11]. Transient post-natal hyperkalemia has been described in patients with KCNJ1 mutations. This is thought to occur due to distal net potassium excretion initially, followed by compensation by other potassium channels in the collecting duct resulting in hyperkaliemia [12].

The genetic mutations underlying this case help to understand the possible pathophysiology of this partial resolution. The *KCNJ1* gene encodes the ROMK channel protein, which is responsible for ATP-dependent potassium recycling in the thick ascending loop of Henle [13]. This patient had two heterozygous mutations in the *KCNJ1* gene creating a compound heterozygous state. A frameshift variant, Glu334Glyfs*35 due to deletion of two base pairs, is predicted to result in premature protein termination 35 amino acids downstream from the deletion. This mutation is located in the cytosolic c-terminus region of the ROMK channel (Fig. 2). If the mutant mRNA transcript does not undergo nonsense-mediated decay, the truncated protein may potentially be translated, and transmembrane component would be intact, allowing potassium transport to occur. The missense variant, Pro110Leu, is located in the extracellular loop of the channel (Fig. 2). This extracellular linker region is known to be less conserved than other regions of the protein suggesting that variants in this region are better tolerated than variants in the transmembrane domain [9].

It has been suggested that the combination of mutations is a crucial determinant in the severity of the phenotype [6, 9, 14]. We hypothesize that our patient’s mild presentation of Bartter syndrome may be a result of having two pathogenic variants outside of the transmembrane domain. The resolution of the hyponatremia may also be explained by the recruitment of additional nephrons due to the physiological postnatal adaptation of renal function [15]. These additional nephrons could compensate for ongoing salt wasting and sufficient compensatory mechanisms may avoid the hypokalemic alkalosis. Interestingly, not all patients with *KCNJ1* mutations develop hypochloremia [14]. We hypothesize that in our patient, only the net potassium and sodium excretion were corrected. Clinically polyuria and polydipsia as well as net calcium excretion remained altered.

This case presents a combination of mutations in the *KCNJ1* gene whose electrolyte abnormalities resolved at 7 weeks of age and only progressed to a mild phenotype. Future presentations of resolving electrolyte abnormalities warrant consideration of Bartter syndrome and specifically mutations on the *KCNJ1* gene. Such patients should be diagnosed with Bartter syndrome type II and remain under the care of a pediatric nephrologist. This case adds to our understanding of the variability of Bartter syndrome and may be used to guide further research into the entity.

## Data Availability

Data sharing is not applicable to this article as no datasets were generated or analysed during the current study.

## Abbreviations

MAGE-D2: Melanoma associated antigen D2
ROMK: Renal outer medullary potassium channel
ACTH: Adrenocorticotropic hormone
